# Response of net primary productivity to vegetation restoration in Chinese Loess Plateau during 1986-2015

**DOI:** 10.1371/journal.pone.0219270

**Published:** 2019-07-10

**Authors:** Xueding Jiang, Wen Shen, Xiaoyan Bai

**Affiliations:** 1 School of Environmental and Chemical Engineering, Foshan University, Foshan, China; 2 Department of Environmental Engineering, School of Environmental Science and Engineering, Guangdong University of Technology, Guangzhou, P. R. China; Tennessee State University, UNITED STATES

## Abstract

Land use and land cover change induced by large scale ecological restoration programs has a significant impact on the terrestrial ecosystem carbon cycle, especially on the net primary productivity (NPP) in arid and semi-arid regions. This study investigated the change in NPP caused by the large-scale ecological restoration in the Chinese Loess Plateau (LPR) region from 1986 to 2015 based on land cover datasets and NPP calculated using the Carnegie-Ames-Stanford Approach model. The results indicated that the annual total NPP exhibited a significant uptrend (*P* < 0.01) throughout the whole vegetation restoration region during the last 30 years, with an annual increase of 0.137 Tg C. A significant abrupt change was detected in 2006 for the annual total NPP series. Over half of the restoration region showed an increase in NPP in the past three decades, however, about 30~40% of the vegetation restoration region exhibited NPP loss before 2006, but subsequently NPP loss was found in only approximately 20% of the study region. Overall, the increase in NPP attributed to the vegetation restoration reached 51.14 Tg C in the past three decades, indicating that these large-scale vegetation restoration programs increased the carbon sequestration capacity of terrestrial ecosystems in the Loess Plateau. The findings of this study improve our understanding of the effects of the green campaign on terrestrial ecosystems.

## Introduction

Land use and cover change (LUCC) caused by human activities fundamentally affects the composition, structure, and function of natural ecosystems [1,2]. The human-driven changes not only essentially convert landscapes, but also alter the carbon storage and flux of terrestrial ecosystems [3,4]. Extreme LUCC can greatly weaken terrestrial carbon sinks, such as urban sprawl [5–7], while rational land management may have a positive impact on the ecosystem carbon sequestration. A series of large-scale vegetation restoration programs have been implemented to prevent environmental degradation in China [8, 9]. Clearly, these vegetation restoration projects have significantly changed the land cover patterns [10, 11]. However, whether these vegetation restoration programs can increase the biological carbon sequestration ability is not clear. Assessing the impact of LUCC caused by vegetation restoration on the ecosystem is critical to understand the change in the ecosystem induced by human activity.

As major components of the carbon cycle, terrestrial net primary productivity (NPP) represents the increase in the total amount of dry organic matter in a vegetation ecosystem through CO_2_ uptake by photosynthesis [12–14]. The NPP is not only the driving force of the carbon cycle, but also a major determinant of carbon sinks as well as a key moderator of ecological processes [15,16]. Therefore, NPP is recognized as a synthetical measure of ecosystem function and a key variable to analyze the impact of LUCC on terrestrial ecosystems [17,18]. Thus, studying the variation in NPP due to vegetation restoration can provide important information on the response of terrestrial ecosystems to intense human activity.

The Loess Plateau region (LPR) in China is well-known for suffering from water shortage, soil erosion, vegetation degradation, and desertification, which has significantly impeded the local the economy and social development [19–21]. To address the environmental problems, a series of vital vegetation restoration programs, such as the Comprehensive Control Project of the Loess Plateau and the Grain to Green Program (GTGP), have been launched in the LPR since the 1980s [22,23]. The GTGP is the largest land-use transition program in the world in recent decades. It involves converting cropland to forest and grassland, closing hillsides to facilitate afforestation and planting tree on barren hills and wasteland [24,25]. By the end of 2012, 24.2 million hectares of both marginal cropland and wasteland had been converted into forest and grassland [21]. The total investment in the GTGP has surpassed 500 billion yuan over the past decades [26]. Generally, to date, vegetation covers have greatly increased, and soil and water loss have been effectively controlled [9,10]. Such large-scale land transformations also enhance the carbon sequestration capacity of the terrestrial ecosystems due to the increase of vegetation productivity [27]. However, these large-scale vegetation restorations in the LPR have also led to some unexpected environmental consequences [28]. As a typical arid and semi-arid region, the annual precipitation in the LPR is relatively low but evapotranspiration is very large, leading to ecological water shortage [29,30]. Therefore, the main vegetation types in this region are typically limited to communities of small halophytic subshrubs and some herbaceous vegetation [31]. Nevertheless, in the short term, many fast-growing trees have been planted in the LPR through vegetation restoration [32]. With the expansion of afforestation, more and more trees started to grow in this region and gradually deplete the groundwater resource [33]. Under these circumstances, the low soil moisture is unable to maintain long-term survival of the man-made forests and grasslands [28,34]. Even worse, the growth of trees consumes large quantities of water, which reduces the groundwater level and prevents native species to survive [35]. The vegetation productivity may be weakened due to the decrease of vegetation cover induced by the available soil moisture decrease. In this case, whether the green campaign can increase the NPP has yet to be determined. The effects of vegetation restoration on NPP in the LPR have received some attention [36–38]. However, overall, studies that specifically focus on the effects of vegetation restoration on NPP are relatively few, and the time period covered was not long enough (approximately only 10 years), making it hard to comprehensively reflect the relation between vegetation restoration and NPP. A thorough and complete understanding of the effects of vegetation restoration on NPP in the LPR is imperative for the local government to develop and implement a more effective policy on vegetation restoration.

Accordingly, this study mainly aimed to assess the impact of vegetation restoration on NPP in the LPR from the late 1980s to 2015. To achieve this goal, we first analyzed the LUCC induced by vegetation restoration from the late 1980s to 2015. We then evaluated the variations of NPP throughout the vegetation restoration region and ultimately assessed the impacts of vegetation restoration on NPP by using the NPP dataset calculated from the Carnegie-Ames-Stanford Approach (CASA) model. The results of this study enhanced our understanding of the effects of the green campaign on the terrestrial ecosystem and can serve as a reference for future ecological policies in the LPR.

## Data and methodology

### Study area

**Fig 1:**
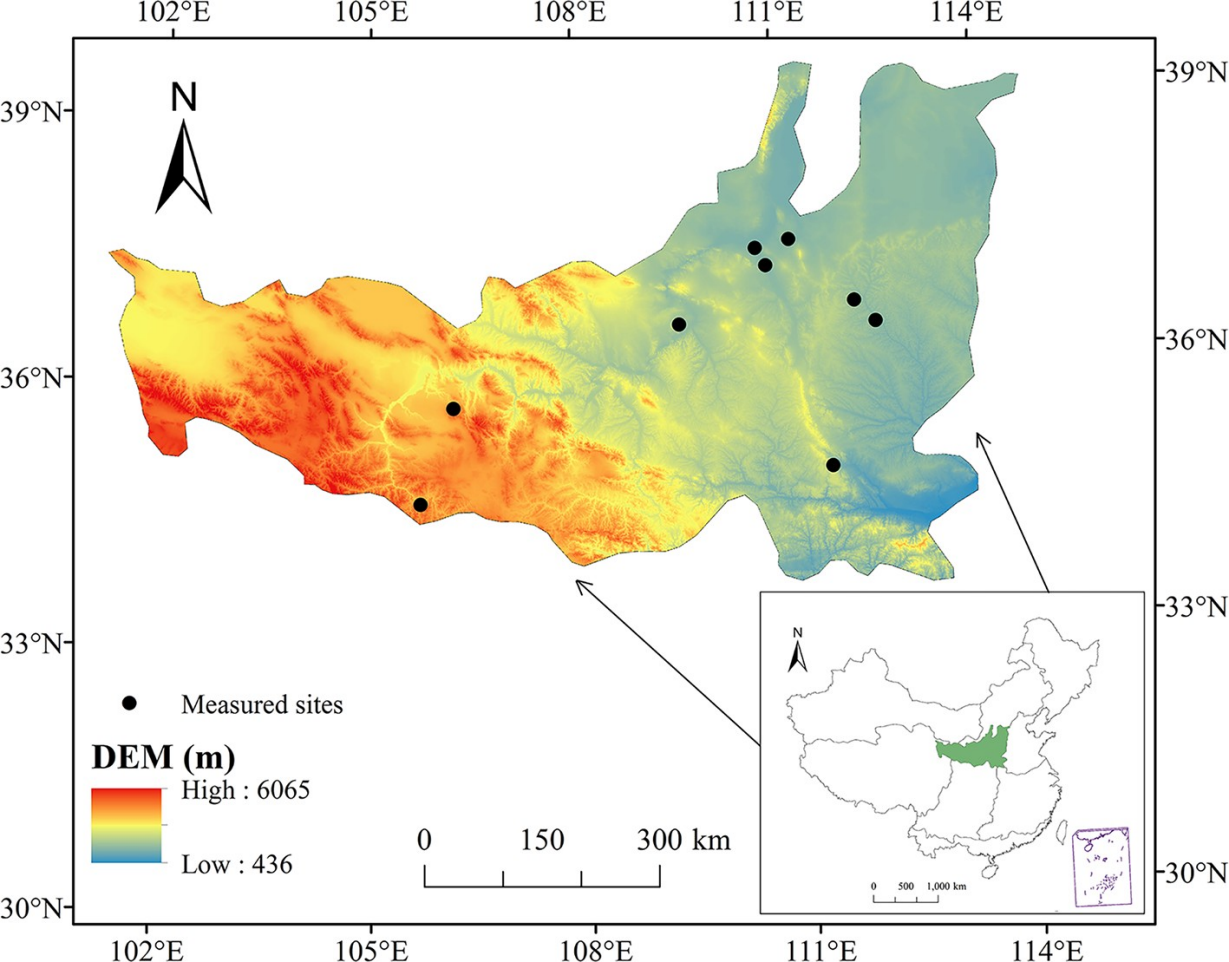
Location of the Loess Plateau and the NPP observation sites.

The LPR is located in the middle reaches of the Yellow River basin, north China (100°54′–114°33′E, 33°43′–41°16′N) and covers an area of about 380,000 km^2^ (Fig 1), with an average elevation of about 1,200 m [10,39]. This region is characterized by an arid and semi-arid climate and has a mean annual precipitation of about 420 mm, with approximately 60–70% of the rainfall occurring during June to September, but the average annual pan evaporation is approximately 1,510 mm [40]. The annual average temperature ranges from 3°C in the northwest to 15°C in the southeast [41]. The surface is covered by highly erodible loess layers. Loess layers are 80–120 m thick on average (300–400 m in typical highland areas) and are the thickest known loess deposits in the world [34]. Serious water shortages, desertification, and soil erosion are the main obstacles for the sustainable economic and social development of the Loess Plateau [19–21, 42]. Significant decrease of vegetation coverage due to anthropogenic disturbances causes severe soil erosion and destroys the local natural ecological environment, leading to the LPR as the most eroded region in the world.

To deal with worsening environmental problems, a series of governance schemes, including optimizing the land use structure and configuration, converting the slopes into terraces, restoring the slope croplands into forests and grasslands, enclosing hillsides and enclosure against grazing, and constructing reservoirs, have been implemented in the LPR since the 1980s [9,42]. These programs have significantly changed LUCC and largely increased the vegetation coverage in the LPR [28].

### Data

The Carnegie-Ames-Stanford Approach (CASA) model was used to simulate the NPP from 1982 to 2015. To run the CASA model, the monthly climatic datasets from 1982 to 2015, including temperature, precipitation, and solar radiation, were used in this study. All these climate data were obtained from the China Meteorological Administration (http://cdc.nmic.cn/home.do). The third-generation Global Inventory Monitoring and Modeling Studies (GIMMS)-3g NDVI dataset with a spatial resolution of 8 km × 8 km from 1982 to 2015 was utilized to calculate the NPP. These datasets were downloaded from NASA (http://ecocast.arc.nasa.gov/data/pub/gimms/). Such NDVI dataset had been corrected to minimize various deleterious effects, such as calibration loss, orbital drift, and volcanic eruptions [43]. Moreover, the NDVI series (GIMMS NDVI 3g) are the only continuous and latest global NDVI datasets that are continually assessed and validated, which provide a reliable observation of the surface vegetation conditions [44]. The NDVI datasets have been widely used to explore the long-term variation of vegetation as well as extensively applied to calculate the global or regional NPP [9,45,46].

The spatial distribution of various vegetation types were obtained from a vegetation map at a scale of 1:1,000,000 [47], which was mainly derived from ground observations. To run the CASA model, the original categories of the vegetation map were reclassified into different vegetation types, including evergreen broad-leaf forest, evergreen needle-leaf forest, deciduous broad-leaf forest, deciduous needle-leaf forest, grassland, cropland, water, rural area, unused land, and urban land.

To acquire the detailed information of the vegetation restoration region in the LPR, the two periods of land use/cover datasets in the late of 1980s and 2015, with a spatial resolution of 1 km × 1 km, were used in this research. The two datasets were respectively obtained from the Resources and Environmental Sciences Data Center (RESDC) and the Chinese Academy of Sciences (http://www.resdc.cn). The RESDC have carried out uniform quality control and integration checking for the datasets. Before developing the dataset, nationwide field surveys were conducted, mostly in the fall for northern China and in the spring for southern China. Land-use situations were surveyed to obtain a great deal of field-investigation records and photographs. The field survey materials and field records were randomly chosen at a 10% ratio to the number of counties to assess the accuracy of the database. The overall accuracy of the land use was above 94.3%, which can meet the requirement of the user mapping accuracy on the 1: 100,000 scale [48,49].

### Method

#### Carnegie-Ames-Stanford Approach (CASA) model

As a satellite-based photosynthetic utilization model, the CASA model has been widely used to evaluate the global and regional NPP [50]. In this model, the NPP is computed as the product of the amount of photosynthetically active radiation (*APAR*) absorbed by green vegetation and light use efficiency that converts the *APAR* into plant biomass growth [51]. The process of the calculation of NPP is described as follows:

NPP(x,t)=APAR(x,t)×ε(x,t) (1)

where *NPP*(*x*, *t*) represents the net primary productivity at a grid cell (x) in the month *t*; *APAR* is the amount of photosynthetically active radiation; *ε* is the light use efficiency of the vegetation.

APAR(x,t)=FPAR(x,t)×S(x,t)×0.5 (2)

where *S* is the incoming shortwave radiation (MJ m^-2^); *FPAR* is the fraction of photosynthetically active radiation absorbed by the vegetation; the constant of 0.5 denotes the ratio of incident photosynthetically active radiation to solar radiation.

$$\varepsilon \left(x,t\right)=\varepsilon_{\max}\times T_{1}\left(x,t\right)\times T_{2}\left(x,t\right)\times W\left(x,t\right)$$ (3)

where *T*_1_ and *T*_2_ account for the effect of temperature stress; *W* accounts for the effects of water stress, and *ε_max_* is the maximum possible efficiency that has been determined for Chinese ecosystems in the research by Pei et al. (2013) [5]. More details on the CASA model can be found in the studies by Potter et al. (1993) [51].

To analyze the impact of LUCC on NPP, we firstly calculated the accumulated area that was returned to forestland and grassland from other land use types during the past three decades. The land datasets in the late 1980s and 2015 were used to represent the LUCC in 1980s and in recent times. Additionally, the LPR has experienced increasing drought frequency and severity due to global warming [52], which may cover up the actual NPP variation induced by vegetation restoration. To avoid the disturbance of climate fluctuations on the NPP, we then used the mean annual temperature, precipitation, and solar radiation from 1982 to 2015 to calculate the NPP. As the original NPP dataset calculated from the CASA model feature a spatial resolution of 8 km × 8 km, to match the spatial scale, we reproduced the NPP datasets calculated from the CASA model with a spatial resolution of 1 km by using resample tool in ArcGIS, [45]. Finally, the NPP variation across the converted region was analyzed to explore the effect of LUCC on NPP.

#### Statistical method

A simple regression model was employed to analyze trends in the annual and seasonal NPP. The Mann-Kendall (MK) analysis was used for abrupt change analysis for the NPP time series. The advantage of the MK test is that the series do not need to follow a certain distribution of the samples, avoiding interference from abnormal values [53]. More detailed information about the MK test can be found in the study by Nasri and Modarres (2009) [54].

## Result

### LUCC induced by vegetation restoration

**Fig 2:**
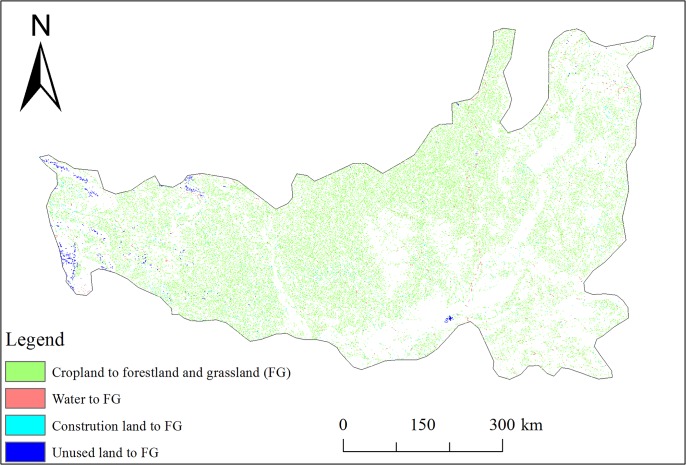
Spatial pattern of the areas that converted to forestland and grassland from other land use in LPR from the late 1980s to 2015. FG represents forestland and grassland.

The spatial patterns of the accumulated area that was returned to forestland, and grassland from other land use types in the LPR in the period from the end of the 1980s to 2015 are shown in Fig 2. According to our statistics, the area converted to forest and grassland during this period was 5.54 × 10^4^ km^2^, which accounted for 14.21% of the LPR total area. As shown in Fig 2, cropland was the main source of new forest and grassland, accounting for 94.58% of the returned area, while water, construction land, and unused land comprised a small proportion of the total conversion area, accounting for only 5.42% of the total transformation area. Overall, the vegetation restoration projects have increased the forest and grassland.

### Validation of the NPP simulation

**Fig 3:**
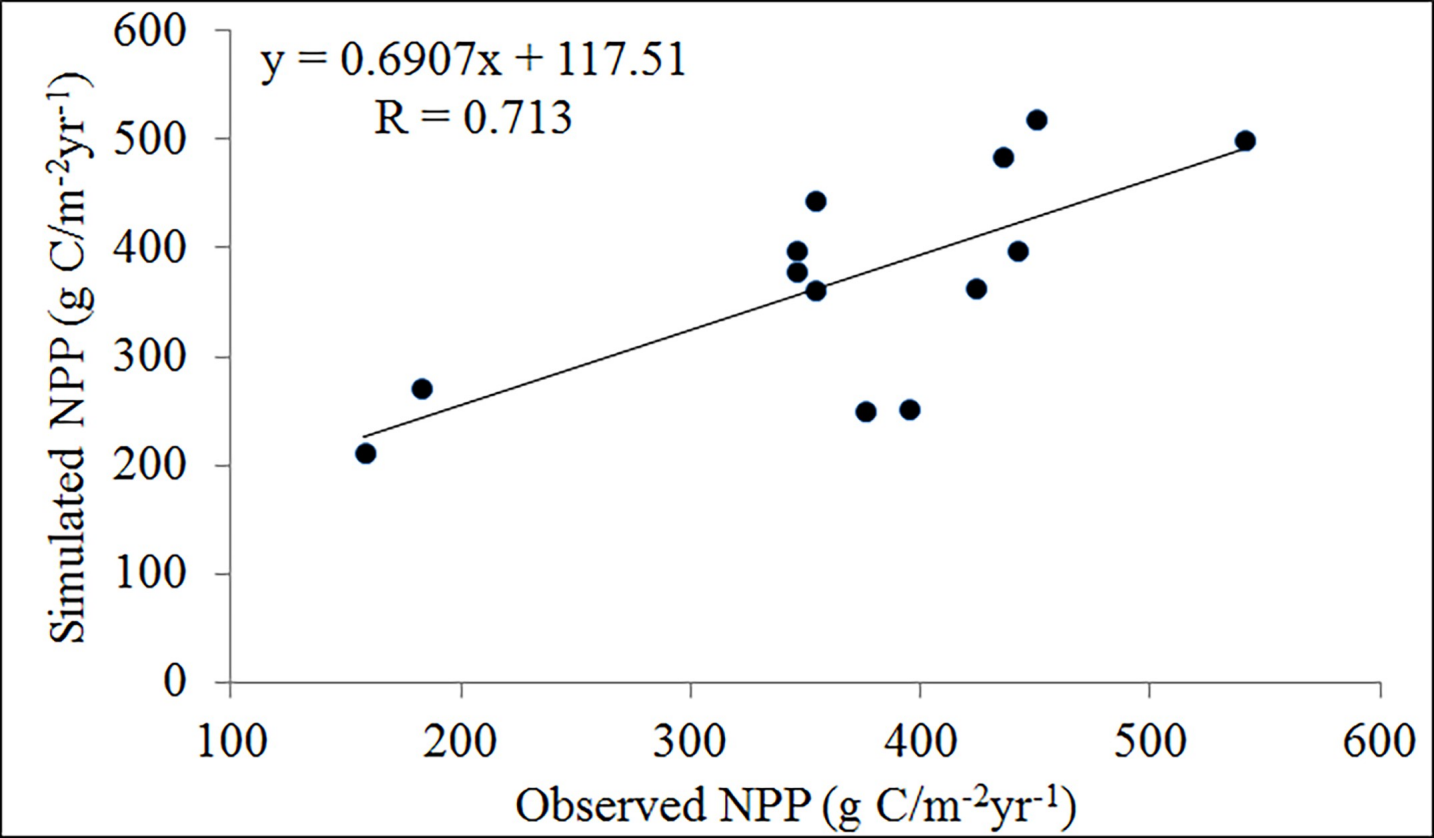
Correlation between the stimulated NPP and measured ground NPP value.

To validate the estimated NPP, we compared the stimulated NPP with the measured ground data and other simulation results. As shown in Fig 3, a good agreement is found between the estimated NPP and the observation-based data, with a high linear correlation (*R* = 0.713, *P* < 0.05).

The simulated NPP values were slightly lower than the measured ground NPP values, which was mainly due to the difference between the spatial resolution of the remote sensing data and the sample size of the measured ground data [55]. According to our statistics, the average annual NPP in the whole LPR was 327.20 g C/m^2^, which is close to the estimate from the MOD17A3 data product (290.07g C/m^2^). The mean value of the NPP in this study was higher than the estimate from the MOD17A3 data product, which is mainly caused by higher NDVI values of MODIS MOD17A3 data product [43]. The higher MODIS NDVI is probably related to the well-known saturation problem whereby NDVI tends to saturate at a high leaf area index, but GIMMS NDVI has a less serious saturation problem due to its different “red” band [56,57].

**Fig 4:**
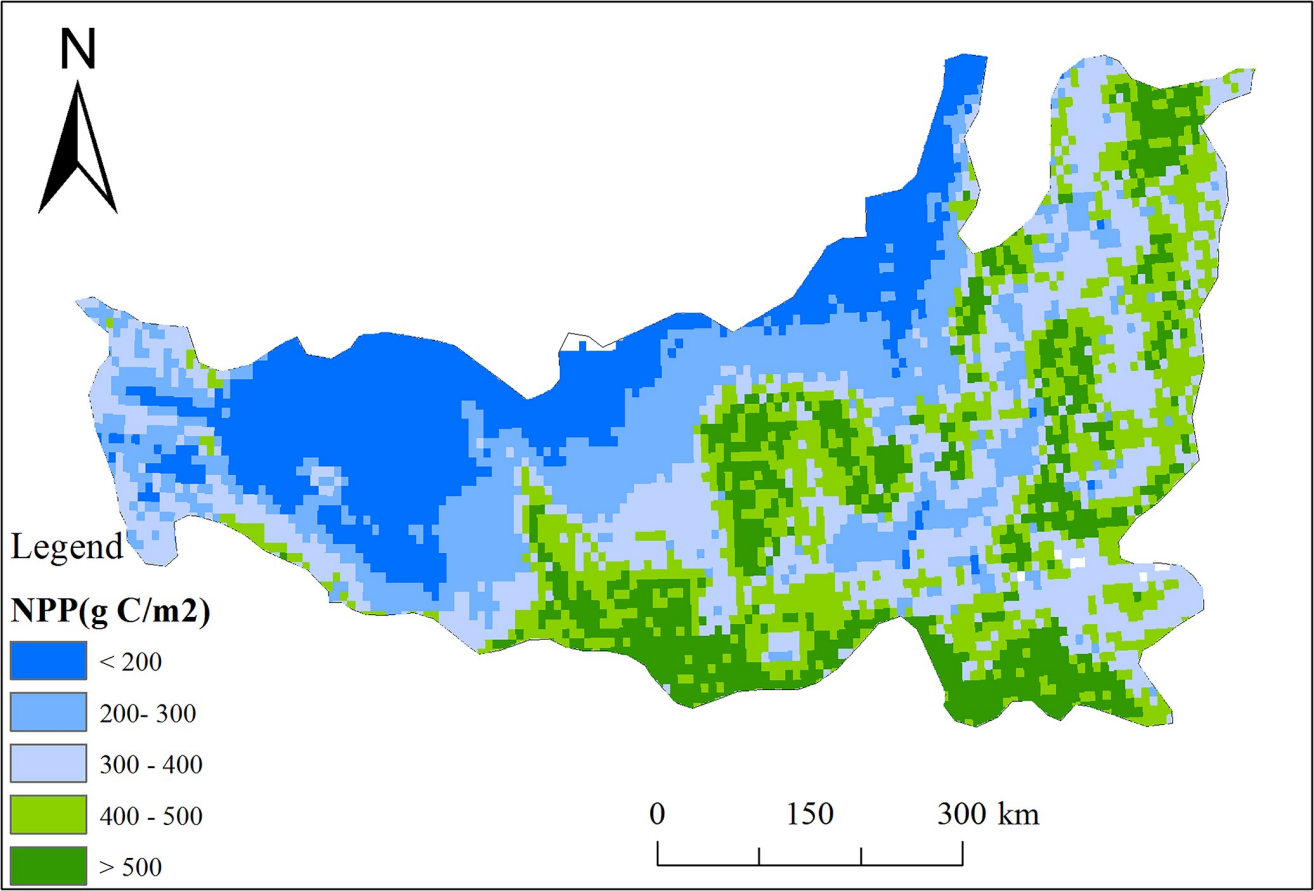
Spatial pattern of average annual NPP from 1986 to 2015 across LPR.

Fig 4 illustrates the spatial pattern of the average annual NPP from 1986 to 2015 throughout the LPR. In general, the NPP displayed gradients decreasing from the southeast to the northwest. Higher annual NPP was observed in the southern edge and central region of the LPR, where the forests and cropland are widely distributed, with NPP higher than 400 gC m^−2^ year^−1^. However, the annual NPP in the northwestern LPR was lower than 200 gC m^−2^ year^−1^, due to the barren land and the low temperature and/or relatively small amount of precipitation (annual precipitation was less than 150 mm) that characterize these areas [38]. The annual NPP in the remaining regions generally ranged from 200 to 400 gC m^−2^ year^−1^. The spatial pattern of the NPP in this region was generally consistent with those of previous studies [5,38,43]. Overall, the results of these analyses implied that our stimulated NPP are reliable in this study.

### Effects of vegetation restoration on the NPP

#### Temporal variation caused by vegetation restoration

**Fig 5:**
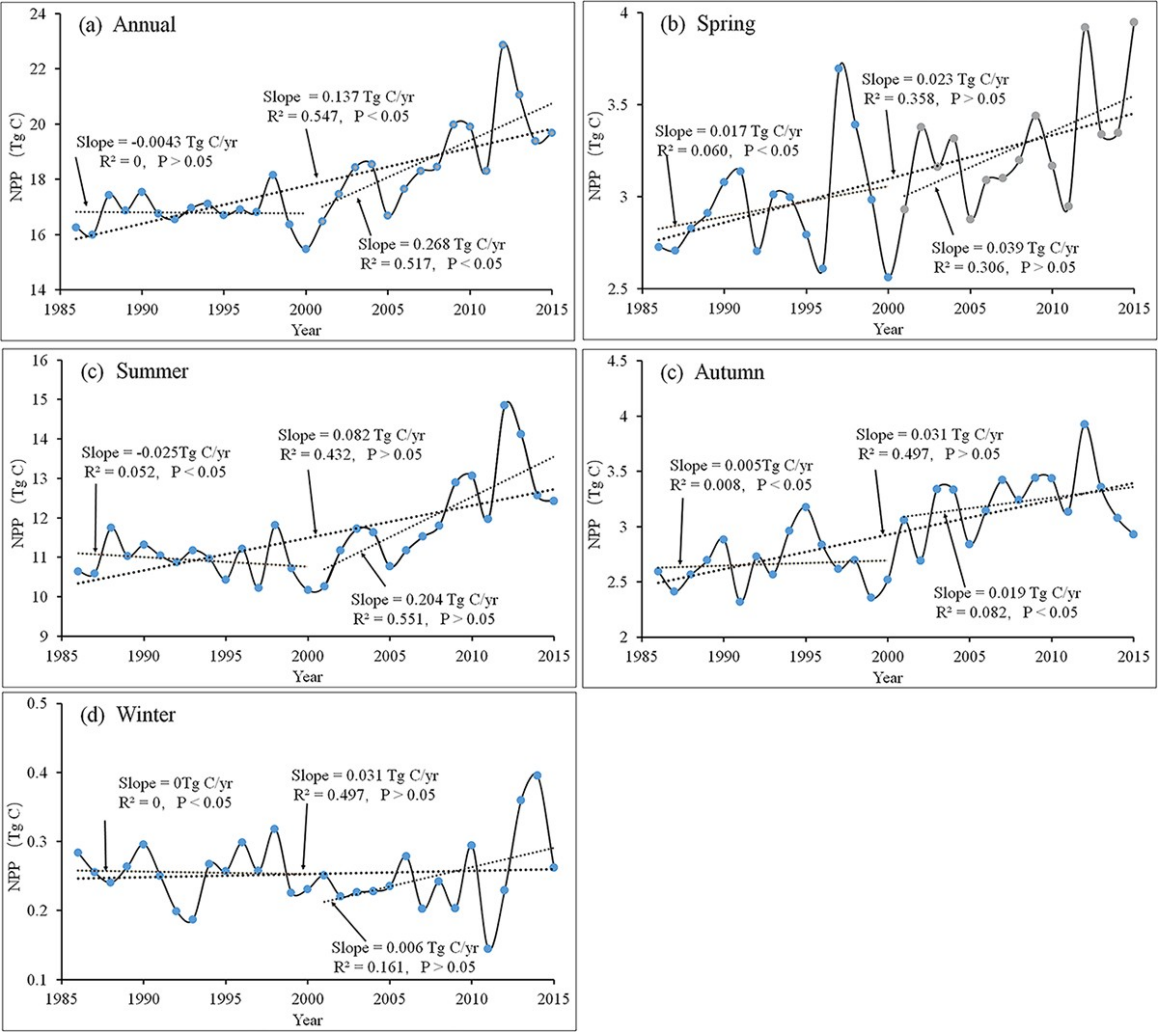
Interannual variations in total NPP at annual and seasonal scale across the vegetation restoration region from 1986 to 2015.

The inter-annual changes in annual and seasonal NPP throughout the vegetation restoration region of the LPR from 1986 to 2015 are shown in Fig 5. At the annual scale (Fig 5A), the annual total NPP exhibited a significant increasing trend (0.137 Tg C yr^-1^, *P* < 0.05) during the last 30 years (Fig 5A). However, the annual total NPP in the ecological restoration region showed different trend during the two sub-periods (1986–2000 and 2001–2015). To our surprise, the annual total NPP decreased slightly from 1986 to 2000, with an annual decrease of 0.004 Tg C yr^-1^. However, after the year 2000, the NPP increased significantly (*R*^*2*^ = 0. 394, *P* < 0.05), with an annual increase rate of 0.286 Tg C yr^-1^. According to our results, the variation of the annual mean NPP shared the similar trend with the annual total NPP. During the past 30 years, the annual increasing rate of the mean NPP was 1.952 gC m^-2^. A mild downtrend was observed from 1986 to 2000 (0.077 gC m^-2^yr^-1^), but after the year 2000, the total NPP increased remarkably (4.780 gC m^-2^yr^-1^).

**Fig 6:**
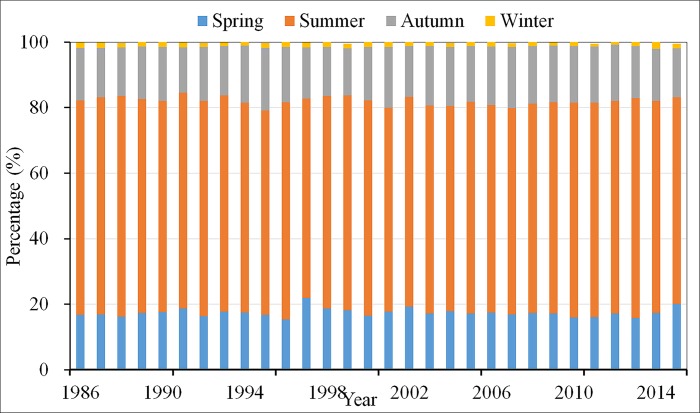
The percentage of seasonal NPP in the among of annual NPP from 1986 to 2015.

The variation in NPP across the vegetation restoration region in the four seasons is illustrated in Fig 5B–5E. Clearly, the total NPP showed a steady increase in spring (0.023 Tg C yr^-1^), summer (0.082 Tg C yr^-1^), and autumn (0.031 Tg C yr^-1^) (*P* < 0.05) over the past 30 years, but no obvious change was found in winter. In the sub-period from 1986 to 2000, the seasonal NPP showed a slight uptrend in spring and autumn (Fig 5B and 5D). In summer (Fig 5C), the NPP showed a large decreasing trend from 1986 to 2000, whereas a significant uptrend (*P* < 0.05) was observed from the year 2000. The NPP showed a slight increase from 2001 to 2015 in autumn and winter (Fig 5D and 5E). In addition, NPP in summer generally accounted for 65% of annual NPP (Fig 6), indicating that the interannual variability in NPP was mainly due to the summer NPP variation.

#### Abrupt change of NPP variation

**Fig 7:**
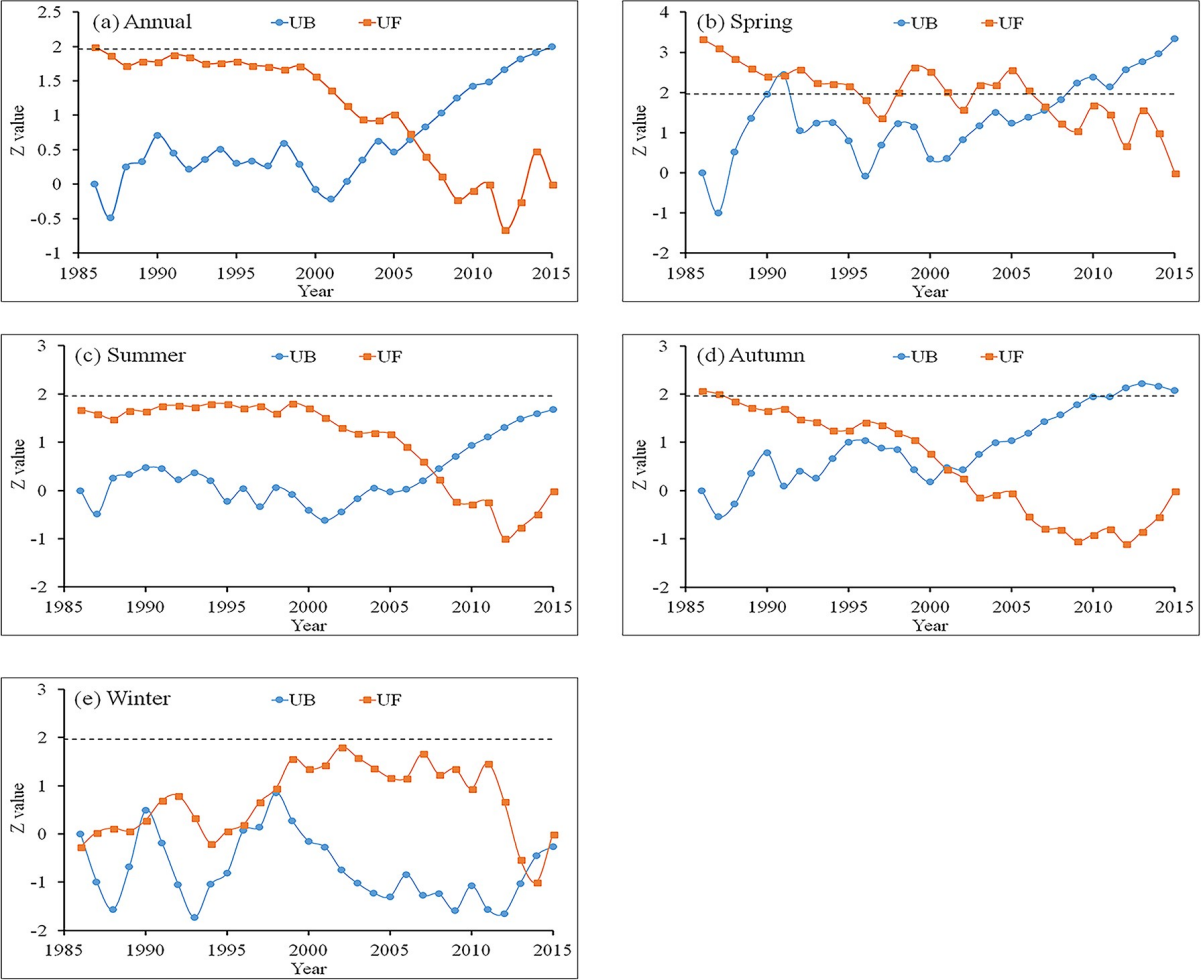
The abrupt change analysis for annual and seasonal NPP. UF and UB represent the statistics of forward and backward sequence respectively.

The abrupt change for annual and seasonal total NPP is shown in Fig 7. For the annual scale (Fig 7A), the UF and UB curves intersected at one time point (2006), indicating that an abrupt change in the annual total NPP series occurred in 2006. Also, the UB curve exceeded the critical value of 1.96, implying that the abrupt change in the annual NPP was significant (*P* < 0.05). In spring (Fig 7B), two abrupt changes were observed in 1991 and 2007, and the abrupt changes were significant as the UB curves passed the critical value. In summer, the UF and UB curves intersected at one time point (about 2007), but none of the curves exceeded the critical value, indicating that the abrupt changes were not significant (Fig 7C). A significant abrupt change was detected in the autumn of 2001 (Fig 7D). More than one abrupt change points were found in winter but none of the change points reached the 0.05 significance level (Fig 7E).

#### Spatial variation caused by vegetation restoration

**Fig 8:**
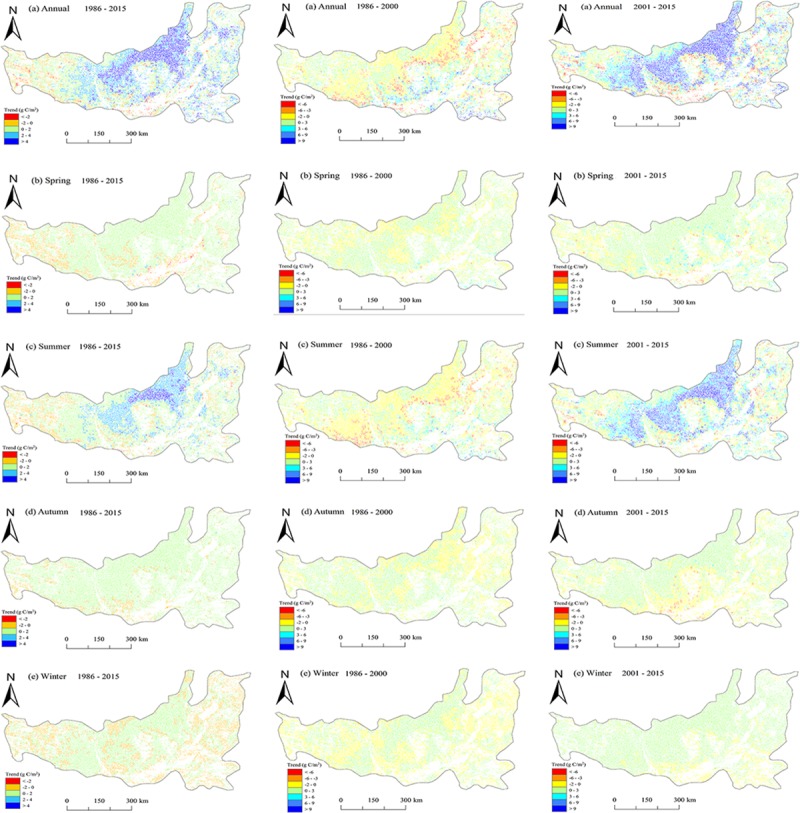
The spatial pattern of NPP trends across the vegetation restoration region at annual and seasonal scales.

The spatial patterns for the annual and seasonal NPP trends throughout the vegetation restoration region are shown in Fig 8. A statistical summary of the annual and seasonal NPP trends during the past three decades and in each sub-period is presented in Table 1. This summary reveals that the annual NPP increased over 87.84% and the restoration region significantly increased over 57.73% (P<0.05) (Fig 8A) during the entire period from 1982 to 2015. The highest increase in the annual NPP mainly occurred in the middle north region, with an annual mean increase of over 4 gC m^-2^yr^-1^, while a large increase (2–4 gC m^-2^yr^-1^) in NPP was concentrated in the west-central area. In contrast, a decrease trend was found in an area mainly located in the west part that was12.16% of the restoration region, with approximately 2.27% of the restoration region decreased significantly (*P* < 0.05). From 1986 to 2000 (Fig 8A), over half of the restoration region showed a decreased trend in the annual NPP, but only 5.35% of it was statistically significant (*P* < 0.05). The large decrease was mainly observed in the north and west-central restoration region, with an annual mean decrease of over 3 gC m^-2^yr^-1^. In contrast, the remaining region (44.89%) exhibited an increasing trend, which mainly occurred in the west and central parts of the study region (Fig 8A). The spatial distribution of the NPP trend in the sub-period from 2001–2015 exhibited a similar spatial pattern to that from 1986 to 2015 (Fig 8A). An increase in NPP was found in an area nearly 80.16% of the restoration region, whereas the remaining region showed a decrease in NPP.

**Table 1:** Statistic summary presenting different trends for annual and seasonal net primary productivity (NPP) acorss the vegetation restoration region in Loess Plateau region (LPR).

Period		Annual	Spring	Summer	Autumn	Winter
1986–2015	Significant decrease (%)	2.27	6.02	2.03	0.56	7.38
	Decrease (%)	9.90	12.57	14.18	9.08	28.36
	Significant increase (%)	30.10	26.51	36.56	30.35	51.67
	increase (%)	57.73	54.90	47.23	60.01	12.48
1986–2000	Significant decrease (%)	2.78	1.48	5.91	0.70	2.01
	Decrease (%)	51.87	34.12	55.18	42.33	47.17
	Significant increase (%)	41.70	58.76	35.82	53.60	49.23
	increase (%)	3.65	5.64	3.09	3.37	1.22
1986–2015	Significant decrease (%)	2.87	3.59	2.06	5.74	2.71
	Decrease (%)	16.97	20.35	17.00	27.03	10.92
	Significant increase (%)	35.52	41.19	36.01	49.05	58.05
	increase (%)	44.64	34.87	44.91	18.18	28.20

In spring (Fig 7B), nearly 81.41% of the study region showed an uptrend from 1986 to 2015, with an annual increase of 0–2 gC m^-2^yr^-1^, and about 54.90% of the restoration region increased significantly. Also, the spatial distribution of the NPP trend in the two sub-periods was generally similar (Fig 8B), with over 64.40% of the study region exhibiting an increase in NPP. During the period from 1986–2015, about 83.79% of the study region showed an uptrend in summer (Fig 8C). For areas showing increased trends in the NPP, nearly half of them are statistically significant (P < 0.05) (Table 1). From 1986 to 2000, a decrease in NPP was observed in 61.09% of the study region, which mainly occurred in the north and west-central of the restoration region, but these regions generally exhibited an increase in NPP from 2000 to 2015 (Fig 8C). The spatial patterns of the NPP trend in autumn and winter are generally consistent (Fig 8D and 8E).

#### NPP increase induced by vegetation restoration

**Fig 9:**
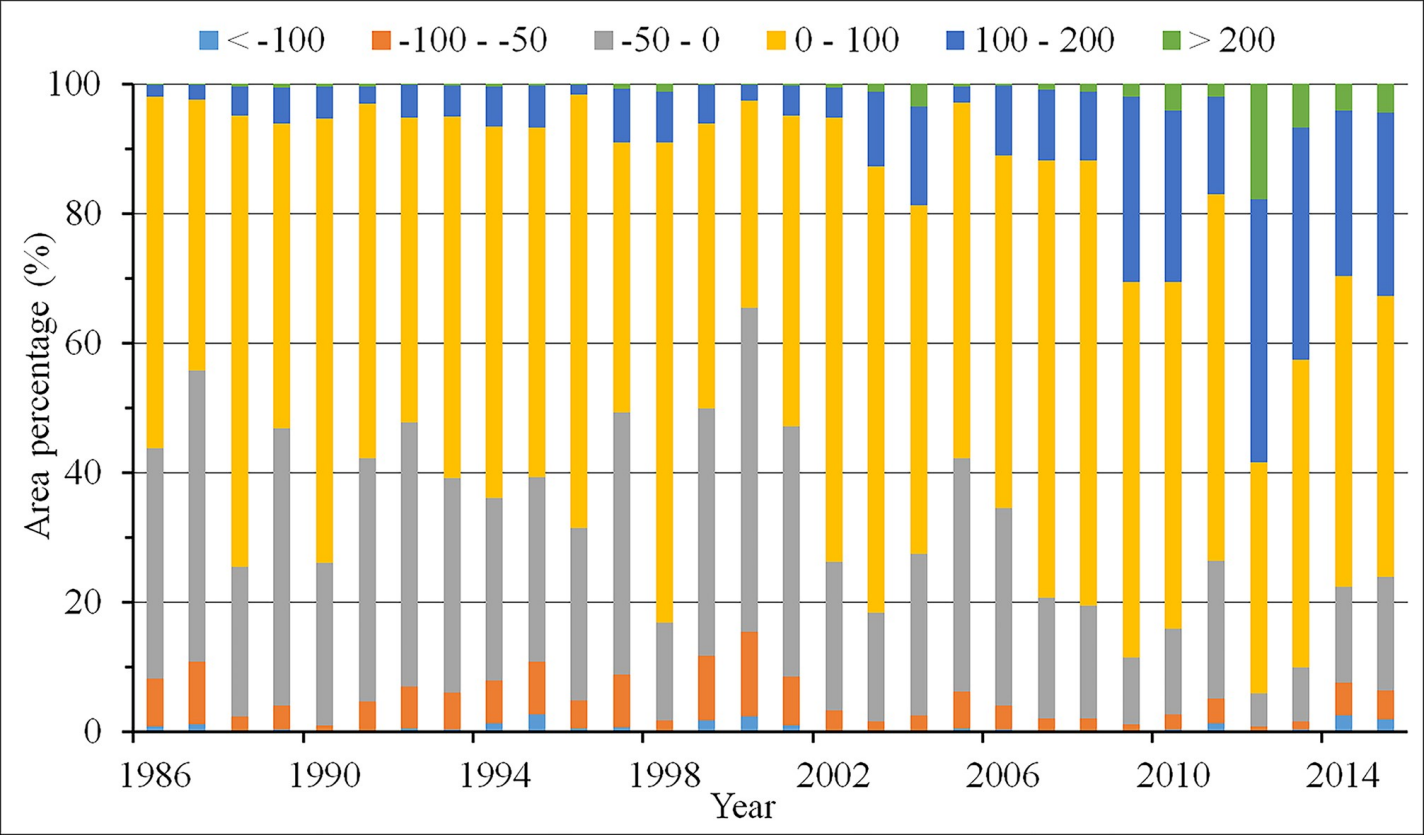
The area percentage of annual NPP difference across the vegetation restoration region from 1986 to 2015. The values with the annual mean NPP minus the annual average NPP from 1982 to 1985 represent the NPP difference.

**Fig 10:**
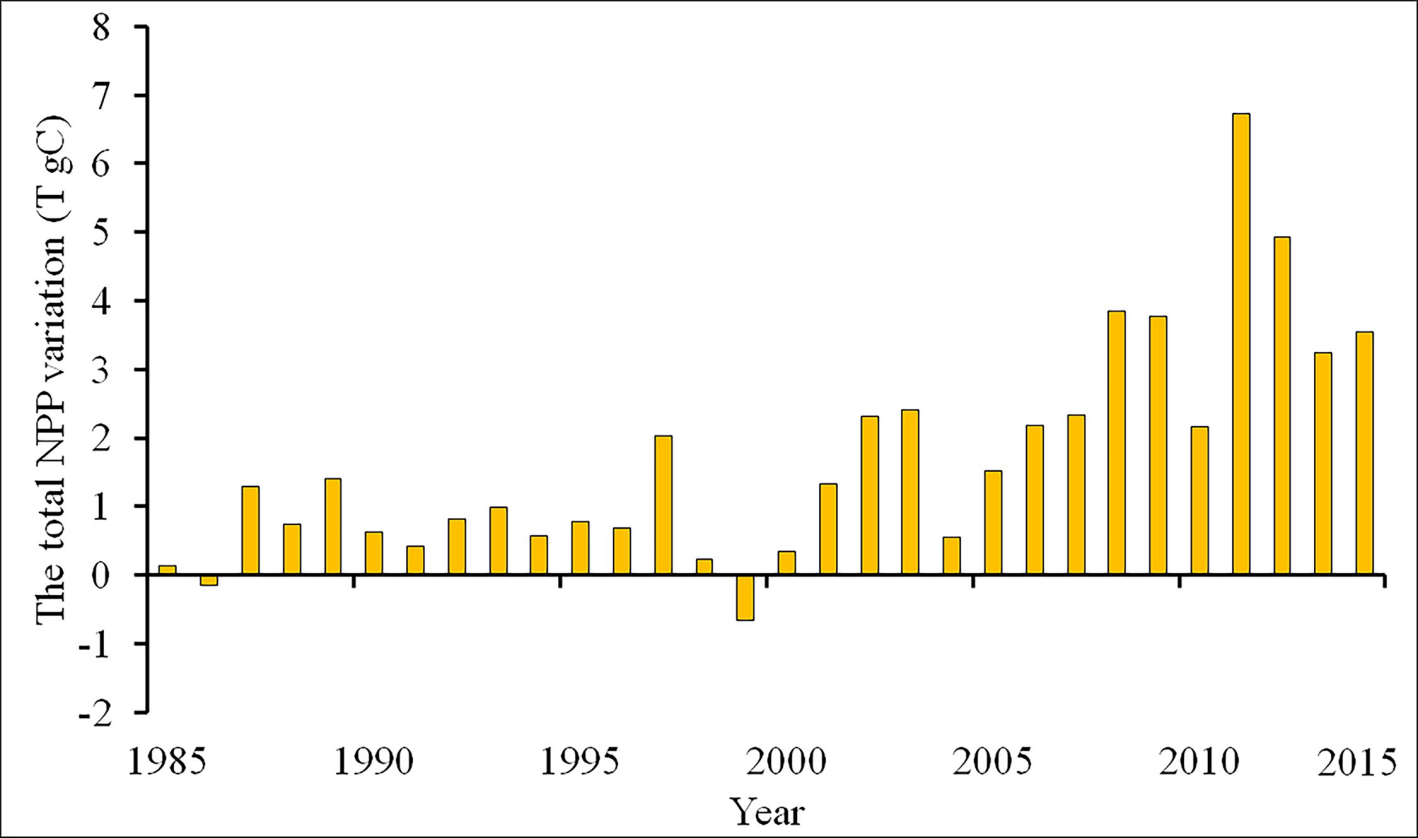
The annual total NPP variation induced by the vegetation restoration from 1986 to 2015. The values with the annual total NPP minus the annual average total NPP from 1982 to 1985 represent the NPP variation; the positive/negative values indicate increase/loss in NPP.

The area percentage with annual mean NPP difference is shown in Fig 9. About half of the restoration region experienced an increase in the annual mean NPP before the year 2000, but after that an increase in NPP was observed across about over 60% of the restoration region, while the remaining region experienced a decrease in NPP. The increase in the annual mean NPP generally ranged from 0 to 100 gC m^−2^year^−1^, while the reduction in NPP mainly varied from 0 to 50 gC m^−2^year^−1^. During the past 30 years, the area where the NPP ranged from 100 to 200 gC m^−2^year^−1^ increased significantly, and the area where the NPP increased by over 200 gC m^−2^year^−1^ also increased largely after the year 2007. In contrast, the area with NPP losses showed a marked reduction (Fig 9). The annual total increase in NPP is shown in Fig 10. In general, the annual NPP exhibited a significant increase during the past three decades. The total increase in NPP was relatively low before the year 2000, with less than 2 Tg C year^−1^. However, the increase was generally larger than 3.5 Tg C year^−1^ after the year 2007. According to our results, the total increase in NPP was 51.14 Tg C from 1986 to 2015.

## Discussion

### Effects of ecological restoration projects on NPP variation

As is known that soil erosion was very serious on the LPR during the past three decades and local people suffered the age-old fragile ecosystem. A series of major ecological restoration projects have been implemented to improve eco-environment [28]. During the past decades, 725 billion RMB (approximately US $100 billion) have been invested to these programs [28,58]. Such effective policies and huge investment produced the instant effect that the vegetation coverage increased significantly [40,59]. As our study indicated, the annual total and mean NPP show a significant increase in the whole vegetation restoration region of LPR during the past three decades. The total increase in NPP in vegetation restoration in the LPR reached up to 51.14 Tg C in the last 30 years. Overall, these ecological restoration projects made significant contributions to carbon sinks and reduced emissions from deforestation and fossil fuel burning [27,60]. The annual carbon sequestration has increased by 132 Tg C/yr as a result of these restoration projects, which is equivalent to 50–70% of the total annual sink from Chinese terrestrial ecosystems and could offset 9.4% of China’s carbon emissions [61]. However, the increasing trend of NPP in the vegetation restoration region was not consistent throughout the past 30 years. The NPP exhibited a slightly decreasing trend before the year 2000, indicating that these programs cannot increase the carbon sinks at first. Planting tree is regarded as an effective measure to mitigate global warming induced by increased greenhouse gas emissions [62,63]. On the other hand, carbon loss from soils may offset the carbon sink effect of planting trees, with decreasing soil organic carbon stocks [64]. Especially during the first 10–20 years after establishing the plantation, soil organic carbon stocks were found greatly decreased due to soil disturbance [65]. Therefore, afforestation may be considerable carbon sources in the early planting trees process due to carbon losses from the soil for tree planting and decreased primary production [64,66].

A significant abrupt change in the annual NPP was detected in about 2006 and the total increase in NPP was relatively large after that. Since the 1970s, the Chinese government has implemented ecological restoration programs, but most of the major programs have been implemented after the year 2000 [67]. Thus, most of the forests in the regions covered by the projects are young, and the forest carbon sink and forest age were growing with logarithmic growth rate [68], indicating that the carbon sinks and vegetation productivity may be relatively low for young-aged forests. The young-aged forests show significant potential to contribute carbon sinks and increase productivity, i.e. the NPP exhibited a significant increase as the forests grew [59]. Moreover, as the forests grow, the increase of the storage of massive amounts of carbon is conducive to the global carbon balance [69]. In summary, the implementation of these ecological restoration projects plays a very important role in mitigating the regional and global climate changes and thus continuous attention should be paid to these projects [27,61].

### Issues induced by ecological restoration projects and the response strategy

It is noteworthy that about 30~40% of the vegetation region experienced a decrease in NPP before the year of 2006. Although the vegetation restoration projects generally contribute to increase the carbon sink and vegetation productivity, these programs also lead to some unexpected problems [33]. To ensure the survival rate and program passing rate early in the implementation of these program, fast-growing trees are most commonly planted in the vegetation restoration region as they achieved the quick and satisfying results in the short-term that are desired by the government [32]. In the arid and semi-arid regions of northern China, the dominant vegetations are small halophytic shrubs, steppe and savanna vegetation, and some herbaceous plants [41]. However, the most important and widely planted tree species across the vegetation region are *Robinia pseudoacacia* and *Pinus tabulaeformis*, which are often unsuitable for afforestation in these regions [10]. Although the annual precipitation is low (< 400 mm), the annual evapotranspiration is high (> 1000 mm) throughout the arid and semi-arid areas in northern China [40,70,71], thus the available soil moisture is not adequate to support the growth of planted trees. As the planted tree begin growing with inadequate water, the groundwater is gradually depleted by the growing trees, which directly depletes the scarce surface water and even lower the water table [72]. Even worse, to achieve a high survival rate, the planted trees density was high across the vegetation restoration region. For instance, the planting density of trees in Dingxi, Gansu Province was about 1,900 plants/ha. However, the recommended suitable planting density in this region is less than 833 plants/ha [73]. The vegetation with high planting density resulted in a large consumption of soil moisture and disturbed the balance of soil moisture and vegetations. Unfortunately, the reduced soil moisture led to a decline in native shallow-rooted vegetation species [10]. Moreover, afforestation with only some species resulted in a situation of simplex tree species and unreasonable forest structure, which readily caused insect and disease problems especially in some dry years [74, 75]. Overall, the vegetation productivity might be reduced due to the improper afforestation in some of the vegetation restoration regions. Fortunately, the problem caused by afforestation has attracted the attention of policymakers, and has been gradually corrected [10]. The decreased NPP of vegetation restoration regions is relatively low after the year of 2006, accounting for about 20% of the study region. Soil erosion and desertification were caused by the complex combinations of natural and human factors, such as climate variation, soil salinization, overgrazing, and unsustainable agricultural practices [76]. An overemphasis on planting trees and shrubs may not be the optimal choice for the ecological restoration of this region [9,33]. Only the combination of social, economic, legal and technical measures can address the environmental problems and achieve sustainable development in ecologically fragile areas. Accordingly, more attention should be paid to the ecological variation throughout the LPR.

## Conclusion

In this study, the variation of NPP induced by the vegetation restoration from 1986 to 2015 in the LPR was analyzed based on land cover datasets using the CASA model. The main conclusions drawn from this study are as follows. The vegetation restoration projects increased the forest and grassland areas in the LPR during the past three decades. The annual mean and total NPP increased significantly throughout the whole vegetation restoration region from 1986 to 2015. In a seasonal scale, the seasonal total NPP increased obviously during the past 30 years except in winter. However, a slight decrease in NPP, except in spring, was found during 1986 to 2000. Spatially, most of the vegetation restoration resulted in increased NPP while the NPP losses were generally scattered in the west and central part of the vegetation restoration region. The annual total NPP series presented a significant abrupt change in 2006. About 30~40% of the vegetation restoration region showed annual mean NPP losses before the year of 2006, but after that annual mean NPP losses were found in only approximately 20% of the study region.

## Supporting information

S1 File Data illustration. (DOCX)
