# Supplementary material for: Response of net primary productivity to vegetation restoration in Chinese Loess Plateau during 1986-2015
Source: PLoS One. 2019 Jul 10;14(7):e0219270. doi: 10.1371/journal.pone.0219270 (PMC6619688; doi:10.1371/journal.pone.0219270)
Supplement: S1 File — (DOCX) [file pone.0219270.s001.docx]

# Response of net primary productivity to vegetation restoration in Chinese Loess Plateau during 1986-2015

Xueding Jiang^1^, Wen Shen^2^, Xiaoyan Bai^2,^*

^1^ School of Environment and Chemical Engineering, Foshan University, Foshan 528000, China; [jiangxueding@fosu.edu.cn](mailto:jiangxueding@fosu.edu.cn);

^2^ Department of Environmental Engineering, School of Environmental Science and Engineering, Guangdong University of Technology, Guangzhou 510006, P. R. China;

[2111707081@mail2.gdut.edu.cn](mailto:2111707081@mail2.gdut.edu.cn) ; [xiaoyanb@](mailto:xiaoyanb@)gdut.edu.cn;

* Corresponding author: [xiaoyanb@](mailto:xiaoyanb@)gdut.edu.cn

# S1. Data

## S1.1 Meteorological Data

The climate dataset employed in this study covers the period of 1982 to 2015, including monthly mean temperature, total monthly precipitation and monthly solar radiation across China. All these data were provided by the Chinese National Metrological Information Center/China Meteorological Administration (NMIC/CMA). For assuring the continuity and consistency, we validated these data by screening and eliminating the suspicious and missing records. In addition, the spatial distributions of these factors were required by the CASA model. As an important interpolation method, Kriging has been widely used when regionalizing various variables at different scales. Thus, in terms of the climate factors from the site-based information, a spatial interpolation of Kriging was applied in this study.

## S1.2 Remote sensing data

The third-generation global inventory monitoring and modeling studies NDVI dataset downloaded from NASA (<http://ecocast.arc.nasa.gov/data/pub/gimms/3g/>) were also used in this study. Such remote sensing data feature a spatial resolution of 8 km × 8km cover the period of 1982 to 2015. The maximum-value composite method was employed to choose the higher value of bimonthly NDVI to obtain the monthly NDVI. In addition, measured ground NPPs from 1989 to 1993 provided by the Chinese Ministry of Forestry were also utilized.

## S1.3 Land use and cover dataset

Two stages of land use/cover datasets in the late of 1980s and 2015 were employed in this study to represent the evolution of land use/cover in Chinese Loess Plateau. The datasets with a spatial resolution of 1 km × 1 km were obtained from the Resources and Environmental Sciences Data Center (RESDC), Chinese Academy of Sciences (<http://www.resdc.cn>). To ensure high-quality and consistent interpretation, RESDC have carried out uniform quality control and integration checking for the datasets. Before developing the dataset, national field surveys were carried out, mostly in the fall for Northern China and in the spring for Southern China. Land-use situations in all provinces, except for Taiwan, were surveyed to obtain a great deal of field-investigation records and photographs. The field survey materials and field records were randomly chosen at a 10% ratio to the number of counties to assess the accuracy of the database. The overall accuracy of the land use was above 94.3%, which can meet the requirement of the user mapping accuracy on the 1: 100,000 scales.
